# Effects of Physical Activity on Amyotrophic Lateral Sclerosis

**DOI:** 10.3390/jfmk5020029

**Published:** 2020-05-06

**Authors:** Grazia Maugeri, Velia D’Agata

**Affiliations:** Department of Biomedical and Biotechnological Sciences, Section of Anatomy, Histology and Movement Sciences, University of Catania, 95123 Catania, Italy; graziamaugeri@unict.it

**Keywords:** amyotrophic lateral sclerosis, physical activity, neuroprotection

## Abstract

Amyotrophic lateral sclerosis (ALS) is a heterogeneous neurodegenerative disease characterized by the loss of upper and lower motor neurons. To date, no resolutive cure is available, and only two Food and Drug Administration-approved drugs are used to treat ALS without a resolutive outcome. In recent years, the study of the beneficial effects of physical activity on health has acquired special relevance. However, the relationship between ALS progression and physical exercise is still a hotly debated topic in medicine. Some studies have suggested higher risks to develop the disease that are associated with practicing intense physical activity, as seen in professional soccer or football players, for example. On the contrary, moderate training has been shown to exert several benefits in ALS-affected patients. Overall, more studies are needed to clarify whether physical activity is helpful or harmful for developing ALS.

Amyotrophic lateral sclerosis (ALS) cases are classified into two types: sporadic ALS (SALS) (~90–95% of ALS) and familial ALS (FALS) (~5%–10% of ALS) form. The SALS etiology is unknown; on contrary, the FALS forms are due to mutations in specific genes, such as chromosome 9 open reading frame 72 (C9ORF72), transactive response DNA binding protein 43 (TARDPB), and Cu/Zn superoxide dismutase 1 (SOD1) [1]. The latter occurs in ~20% of FALS cases [2], and over the years more than 160 mutations in SOD1 gene have been found, including G93A, A4V, H46R, and D90A. 

The pathogenetic mechanism involved in motor neuron degeneration during ALS is not understood yet. However, it seems that several insults contribute to MN damage, including excitotoxicity, increased oxidative stress, neurofilament aggregation, alteration of axonal transport, protein misfolding/aggregation, and activation of microglia leading to inflammation [3,4,5,6,7].

A specific treatment for ALS-affected patients has not been found yet. To date, riluzole and edaravone have been approved by the Food and Drug Administration to treat ALS [8,9]. However, these drugs provide modest benefits limited to some patients.

Physical activity represents a useful means in which to improve general health [10]. It positively affects cardiovascular and neuromuscular systems by improving respiratory, heart, and circulatory function and increasing the hypertrophy and strength of muscle fibers [11,12].

The beneficial effects of physical exercise are also relevant in several pathologies, including cardiovascular and pulmonary diseases, metabolic disorders (e.g., type 2 diabetes and obesity), muscle, bone, and joint diseases, cancer, and neurodegenerative disease [13,14,15,16,17].

Until now, the role of physical exercise in ALS pathology has been controversial. Some epidemiological studies have suggested that people practicing intense physical activity, like professional soccer or football players, have a higher risk of developing the disease [18,19,20,21]. This could be due to hard and prolonged exercise inducing inflammation, oxidative stress, and glutamate excitotoxicity in MNs, all mechanisms involved in their degeneration. On the other hand, some evidence has shown the protective effect of mild physical exercise in ALS. Studies performed on G93A-SOD1 transgenic mice have demonstrated that moderate running-based training increased their survival rate by delaying the onset of disease [22,23]. On the contrary, high endurance exercise seems to have detrimental, if any, effects in these animals [24]. Therefore, the intensity of training represents a crucial aspect for consideration in order to ensure the benefits of physical activity. Neuroprotective effects have also been observed in ALS mice after swimming training. This type of physical activity reduced MN loss due to the modulation of different trophic factors, such as insulin-like growth factor 1 (IGF-1) and brain-derived neurotrophic factor (BDNF) [25]. In accordance, IGF-1 and BDNF are key players of different neuronal functions in the central nervous system, including synaptic density, neurogenesis, and neuron differentiation and survival [26,27,28]. Other studies support the hypothesis that physical activity may also play a beneficial role in ALS patients. Ribeiro et al. [29] showed cessation of muscle cramps in three patients performing yoga therapy, which was composed of breathing and relaxation exercises. Different types of exercise training, including stretching, resistance, or concurrent training, display positive effects on the quality of life by improving muscle strength and cardiorespiratory function [30,31]. In particular, Drory et al. [32] showed that a moderate daily exercise program decreased the loss of motor function, fatigue, and pain of ALS patients after 6 months. Improvements in motor function and quality of life were also found in ALS patients performing daily stretching and resistance exercises [33]. However, the limited number of patients and the small period considered limit the ability of these studies to support the hypothesis that moderate physical exercise is beneficial for ALS patients. In accordance, Dal Bello-Haas and Florence [34], in an update of a review first published in 2008, declared that “there is a complete lack of randomised or quasi-randomised clinical trials examining aerobic exercise in ALS population”.

In conclusion, the role of physical activity during development of ALS is still a debated topic. Future studies are needed to clarify whether it is helpful or harmful for disease progression.
